# Evaluation of the Alkali–Silica Reaction Potential of Korean Aggregates: Experimental Insights and Mitigation Strategies for Concrete Durability

**DOI:** 10.3390/ma18143373

**Published:** 2025-07-18

**Authors:** Chul Seoung Baek, Byoung Woon You

**Affiliations:** Aggregate Resource Research Laboratory, Korea Aggregates Research Institute, Songpa-gu, Seoul 05621, Republic of Korea; ybw@ark.re.kr

**Keywords:** aggregates, alkali–silica reaction, soundness, acid producing, weathering index

## Abstract

The alkali–silica reaction (ASR) is an important mechanism of concrete deterioration, whereby reactive silica in aggregate interacts with cement alkalis to form expanding gel, which compromises the structural integrity of the concrete. Although the Republic of Korea has historically been classified as a low-risk region for ASR due to its geological stability, documented examples of concrete damage since the late 1990s have necessitated a rigorous reassessment of local aggregates. This study evaluated the ASR potential of 84 aggregate samples sourced from diverse Korean geological regions using standardized protocols, including ASTM C 1260 for mortar bar expansion and ASTM C 289 for chemical reactivity, supplemented by soundness, acid drainage, and weathering index analyses. The results indicate expansion within the range of 0.1–0.2%, classified as potentially deleterious, for some rock types. In addition to ASR reactivity, isolated high anomalies (e.g., high soundness, acid producing, and weathering) suggest the existence of other durability risks. Consequently, while Korean aggregates predominantly have a low ASR reactivity, the adoption of various validated ASR tests as a routine test and the integration of supplementary cementitious materials are recommended to ensure long-term concrete durability, highlighting the need for sustained monitoring and further investigation into mitigation strategies.

## 1. Introduction

The alkali–silica reaction (ASR) is an important chemical process that undermines the long-term durability of concrete structures globally. This process occurs when alkali cations, predominantly sodium (Na^+^) and potassium (K^+^) derived from the pore solution in the cement matrix, interact with reactive siliceous phases within aggregates under high pH. The resultant formation of an expanding alkali–silica gel, if there is sufficient moisture, causes volumetric expansion, micro-cracking, and progressive deterioration of the concrete matrix [1].

Fanijo et al. [2] highlighted that ASR is a durability challenge, noting its capacity to impair the mechanical properties of concrete, such as the modulus of elasticity and tensile strength, as also corroborated by Mohammadi et al. [3]. The kinetics and severity of ASR are controlled by multiple factors, including moisture availability, ambient temperature, and the mineralogical composition and granulometry of aggregates, with optimal reactive particle sizes (i.e., the Pessimum effect that accelerates ASR) being variable for different aggregate types [4,5].

Internationally, ASR has been the subject of significant research, leading to the development of standardized testing protocols and mitigation strategies that aim to minimize its effects on structural integrity [6,7]. In South Korea, ASR has historically been regarded as being of negligible concern, based on the presumed low reactivity of Korean aggregates due to their geological provenance [8]. This perception persisted until the late 1990s, when empirical evidence of concrete degradation linked to ASR became evident, particularly in infrastructure such as highways under the control of the Korea Expressway Corporation [9]. These instances of degradation suggest that localized environmental conditions or evolving construction methodologies exacerbated the susceptibility of Korean concrete to ASR, thereby necessitating a reassessment of prior assumptions [10]. These developments highlight a need for a systematic investigation of the reactivity of aggregates used in Korean construction. Using a range of experimental methodologies, comprising chemical reactivity assessments (e.g., KS F 2545, the Korean standard test method following ASTM C 289), mortar bar expansion tests (e.g., ASTM C 1260), [11,12,13], and evaluations of soundness, acid drainage parameters Net Acid Generation (NAGpH) and Net Acid Producing Potential (NAPP) [14], and weathering indices (Chemical Index of Alteration (CIA) and Chemical Weathering Index (CWI)), this study assessed the susceptibility of the aggregates to ASR. The objectives were to empirically determine the reactivity of Korean aggregates and to develop scientifically substantiated mitigation strategies for enhancing concrete durability.

This research builds on other studies of Korean aggregates, such as that by Yun et al. [15], and incorporates perspectives from the global studies of Rajabipour [16] and Shi and Lothenbach [17]. The theoretical framework for ASR mitigation, as developed in this study, is based on established mechanisms, including the imbibition pressure, ion diffusion, crystallization pressure, and electrical double-layer repulsion, which collectively contribute to the expanding behavior of ASR gels [16]. These mechanisms highlight the importance of controlling the alkali contents and moisture ingress to prevent gel formation and subsequent damage [7].

By investigating the ASR potential of Korean aggregates, this study attempted to address a gap in our understanding of the durability of concrete infrastructure in Korea [18]. The findings will inform material selection and treatment protocols in construction practice, potentially involving the use of supplementary cementitious materials (SCMs), such as metakaolin [19], or the regulation of alkali contents [20]. Furthermore, in light of the increased incorporation of cataclastic granites and recycled aggregates in concrete production [5,21], which leads to increased ASR risks due to reaction heterogeneity, this study established a basis for sustainable concrete technologies in Korea.

## 2. Materials and Methods

### 2.1. Sample Collection and Preparation

A total of 84 aggregate samples were collected from diverse geological regions across South Korea, representing the range of materials used in construction projects nationwide. These samples were collected between 2021 and 2024 through resource surveys conducted by the Ministry of Land, Infrastructure and Transport [8]. The sampling sites comprised 18 zones, including 3 southern coastal localities (Geoje, Gwangyang, and Gangjin), 2 southern inland regions (Gokseong and Muju), 8 central inland areas (Eumseong, Jeungpyeong, Gunwi, Nonsan, Icheon, Chungju, Dongducheon, and Yangpyeong), 4 western coastal regions (Yeongdeok, Buan, Ansan, and Incheon), and 1 northern inland region (Pocheon). This geographical range ensured the inclusion of various lithologies, such as granite, gneiss, schist, phyllite, tuff, shale, and marble. The rock types are summarized in Table 1, which highlights the predominance of igneous rocks (46 samples), followed by sedimentary rocks (22 samples), metamorphic rocks (11 samples), and volcanic rocks (5 samples).

Following collection, the aggregates were mechanically crushed to a maximum particle size of 4.5 mm, sieved to ensure uniformity, and subdivided into representative aliquots in accordance with standardized protocols. This preparation minimized the variability in ASR test results associated with particle size differences, which influence ASR reactivity [4].

### 2.2. Testing Methods

**Figure 1:**
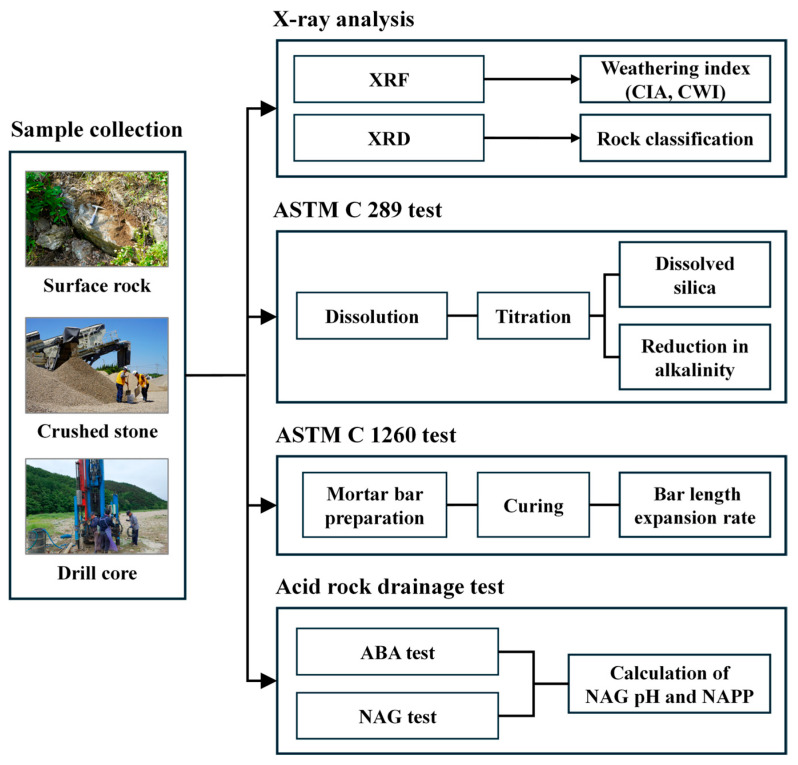
Experimental flowchart for ASR potential evaluation of aggregates.

Figure 1 illustrates the experimental flowchart used in this study to evaluate the ASR potential of aggregates. Various experimental techniques were used to assess the ASR potential of the collected aggregates, applied according to Korean and international standards. The chemical reactivity was evaluated using KS F 2545: Test Method for Potential Alkali–Silica Reactivity of Aggregates (Chemical Method), which is a Korean standard analogous to ASTM C 289: Standard Test Method for Potential Alkali–Silica Reactivity of Aggregates (Chemical Method), which quantifies the dissolution of silica in an alkaline medium and is designed to measure the reactive silica content of aggregate. Although ASTM C 289 was withdrawn internationally in 2016 due to potential inaccuracies due to interference by minerals such as talc or zeolite, it remains a sanctioned method in Korea and was thus used to provide a comparison with previous Korean studies. Mortar bar expansion tests were conducted by following ASTM C 1260: Standard Test Method for Potential Alkali Reactivity of Aggregates (Mortar-Bar Method) and RILEM AAR-2: Detection of Potential Alkali-Reactivity—Accelerated Mortar-Bar Test Method for Aggregates protocols [22], involving the preparation of mortar specimens using the test aggregates, exposure to a 1 N NaOH solution at 80 °C for 14 days, and measurement of length changes to determine the expansion potential. While these methods are effective for the rapid detection of ASR-induced expansion, multiple recent studies have reported potential inaccuracies associated with their use.

Supplementary analyses included the soundness test conducted using KS F 2507 (Test Method for Soundness of Aggregates by Use of Sodium Sulfate) [23], which evaluates aggregate durability through sodium sulfate exposure. In addition, acid drainage tests were performed using NAPpH and NAPP to evaluate the environmental stability and potential chemical reactivity under acidic conditions. Weathering indices (CIA and CWI) were determined by X-ray diffraction (XRD) and X-ray fluorescence (XRF) analyses to characterize the degree of mineral alteration, which provides indirect insights into silica reactivity.

The mineralogical composition of samples exhibiting alkali–silica reactivity was further characterized using XRD and XRF analyses. XRD analysis was employed for rock classification and the identification of silicate minerals, considering that it cannot detect amorphous silica, while XRF analysis was utilized to assess the SiO_2_ content and identify mineralogical indicators associated with weathering. All these tests followed standardized conditions to ensure data reproducibility, with sample preparation and analysis conducted in triplicate where feasible. XRD and XRF analyses were conducted a X’Pert MPD (Philips, Almelo, The Netherlands) and a Lab Center XRF-1700 (Shimadzu, Kyoto, Japan), respectively, both of which are operated by the Korea Institute of Geoscience and Mineral Resources.

### 2.3. Data Analysis

Data from the tests were analyzed to classify the ASR potential of the aggregates. For ASTM C 1260 and RILEM AAR-2, the expansion thresholds were adopted from established guidelines: expansion of <0.10% at 14 days was deemed innocuous, while expansion of 0.10–0.20% indicated potential physical degradation, and expansion of >0.20% indicated deleterious reactivity, with intermediate values suggesting potential reactivity and thereby requiring further scrutiny (ASTM C 1260). Chemical reactivity data from ASTM C 289 and KS F 2545 were interpreted using silica dissolution thresholds, although adjustments were made to account for known limitations of ASTM C 289, such as false-positive results arising from non-siliceous constituents.

Soundness values were evaluated against a threshold of 12% mass loss, beyond which aggregates with aggregates at or near this value considered susceptible to physical degradation that could intensify the effects of ASR. Acid drainage parameters (NAGpH and NAPP) were used to identify aggregates with anomalous chemical behavior; i.e., NAGpH < 4.5 or positive NAPP values indicate acid generation capacity and potentially heightened reactivity.

Weathering indices (CIA and CWI) were compared with reference ranges for fresh and weathered rocks, with higher values indicating greater alteration and possible susceptibility to ASR. The results were cross-referenced with mineralogical data to correlate the reactivity with specific lithologies (e.g., quartz-rich granite versus phyllite).

Statistical analysis, including mean and variability assessments, was undertaken to validate the findings, although the limited replication of some tests constrained the application of advanced statistics. Our results were benchmarked against international [16] and Korean [15] studies to contextualize the ASR potential within a broader framework.

## 3. Results

### 3.1. Aggregate Characterization

The analyzed samples include granite, gneiss, schist, phyllite, tuff, shale, marble, andesite, and diorite, as shown by the subset of 21 samples with confirmed ASR potential (Table 2). Table 3 presents the chemical compositions of the tested samples, while Table 4 integrates the results of all ASR-related properties, enabling a comprehensive evaluation of reactivity trends. These datasets form the basis for the subsequent analysis and discussion of ASR behavior across different lithological categories.

Granite is the predominant rock type, followed by gneiss and schist, reflecting their widespread use in Korean construction. XRD analysis confirmed the presence of quartz as a primary constituent in most samples, along with variable proportions of feldspars, micas, and accessory minerals, although specific quantitative mineral abundances are not reported. The XRF data corroborate the siliceous nature of the aggregates, with SiO_2_ contents of 60–80% derived from reactivity tests, which is a prerequisite for ASR susceptibility (Table 3). The CIA and CWI values vary widely (CIA = 1.3–76.2; CWI = 1.3–98.2; Table 4), indicative of variable degrees of mineral alteration between the samples.

### 3.2. Assessment of ASR Potential

A total of 21 samples were identified as having potentially deleterious expansion based on the two ASR potential assessment tests (Table 4). In the chemical reactivity test, expansion in 17 samples was classified as either potentially deleterious or deleterious. In the mortar bar expansion test, expansion in 19 samples was classified as potentially deleterious, whereas no samples showed deleterious expansion.

**Figure 2:**
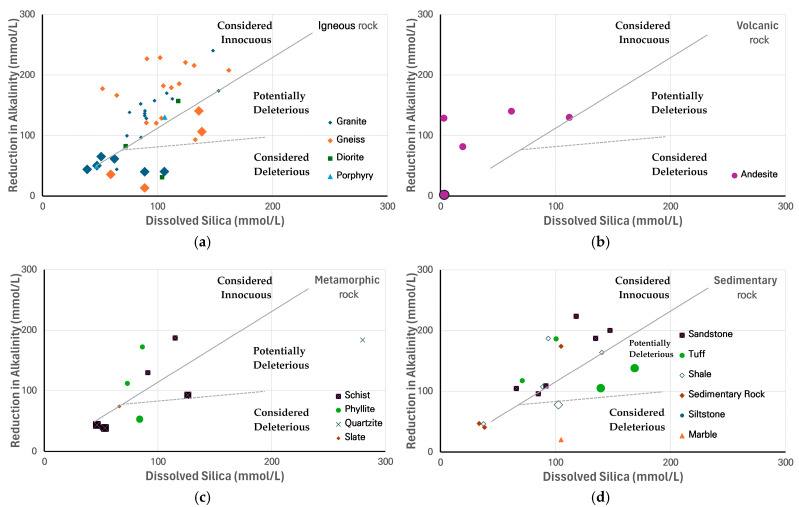
ASR test results showing dissolved silica concentrations (Sc) and reduction in alkalinity (Rc) for various rock types: (**a**) igneous, (**b**) volcanic, (**c**) metamorphic, (**d**) sedimentary rocks.

Figure 2 presents the overall ASR test results across various rock types, showing ASR reactivity in igneous, volcanic, metamorphic, and sedimentary rocks [9]. Although the ASR reactivity graph of ASTM C 289 is originally plotted using logarithmic values, the tested samples exhibited low Sc and Rc values, leading to the data being represented in a linear format. The graph includes a total of 84 samples markers, among which the large markers represent 19 samples exhibiting expansion of 0.1% or more in the mortar bar test. ASR potential was identified in six granite samples from six locations and in four gneiss samples from four locations. Considering that large quantities of the aggregate used in Korea are derived from granite and gneiss, these findings indicate that ASR testing should be conducted based on the intended use of the concrete. However, many private construction projects in Korea omit ASR testing.

**Figure 3:**
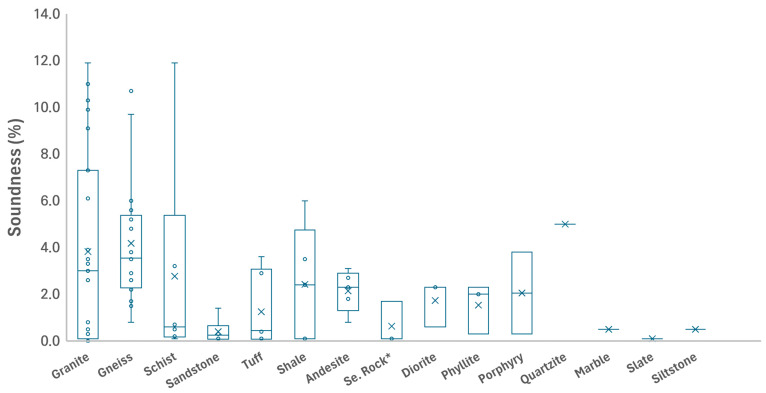
Box plots of soundness values from the KS F 2507 test for different rock types, highlighting the variability in their physical durability. (Small circles (°) represent individual data points, crosses (×) indicate the mean values, and horizontal lines (—) indicate the median values).

Figure 3 presents the soundness values for the different rock types, with the gneiss and schist samples exhibiting greater variability than the granite samples. Supplementary tests provided additional insights into the aggregate stability. Soundness values, assessed via KS F 2507, ranged from 0.1% to 11.9% mass loss, with most samples (e.g., 23BA-MG03 and 23NS-MG06) exhibiting exceptional durability (<2%), while outliers such as samples 21ICN-MG08 (schist; 11.9%) and 21AS-MG04 (gneiss; 11%) approached the 12% threshold indicative of potential physical degradation.

**Figure 4:**
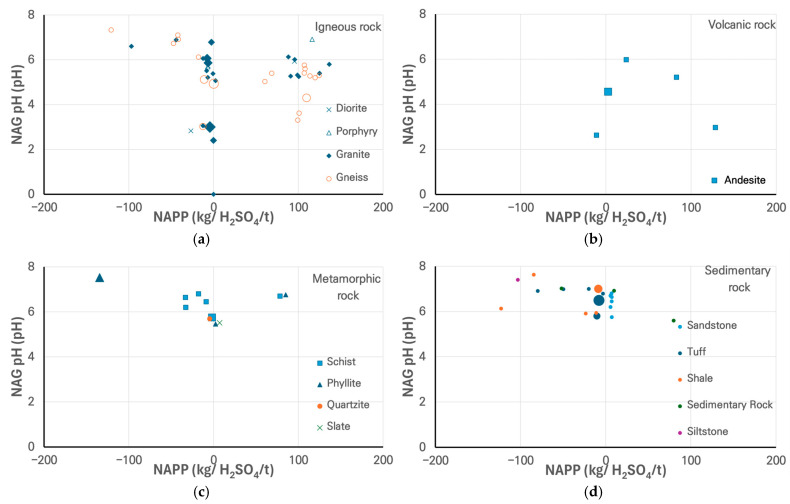
Acid drainage parameters (NAGpH versus NAPP) for rocks, showing their chemical stability: (**a**) igneous, (**b**) volcanic, (**c**) metamorphic, (**d**) sedimentary rocks.

Figure 4 presents the acid drainage results. Rocks that are likely to generate acid due to the oxidation of sulfide minerals have difficulty maintaining the high pH required for silica dissolution, thereby inhibiting ASR activity. Samples represented by larger symbols were identified as being potentially reactive in the mortar bar test. Consequently, most samples considered potentially deleterious due to ASR are located within regions characterized by low or negative absolute NAPP values. For samples exhibiting ASR reactivity, NAGpH values varied from 2.40 to 8.14 (Table 4), with most values above the 4.5 threshold for acid generation risk, whereas excluding one highly weathered gneiss sample, the remaining nine samples have a narrow range of NAPP values, from −12 to +5 kg H_2_SO_4_/t. The volcanic, metamorphic, and sedimentary rocks show similar trends to the igneous rocks. One exceptional sample shows a lower NAPP value than the other sample (22PC-MG24) due to the presence of a component (CaO) with high acid reactivity among its primary constituents. Additionally, the values (–8.14, –888.4) fall well outside the range presented in the table and are therefore omitted from Figure 4.

**Figure 5:**
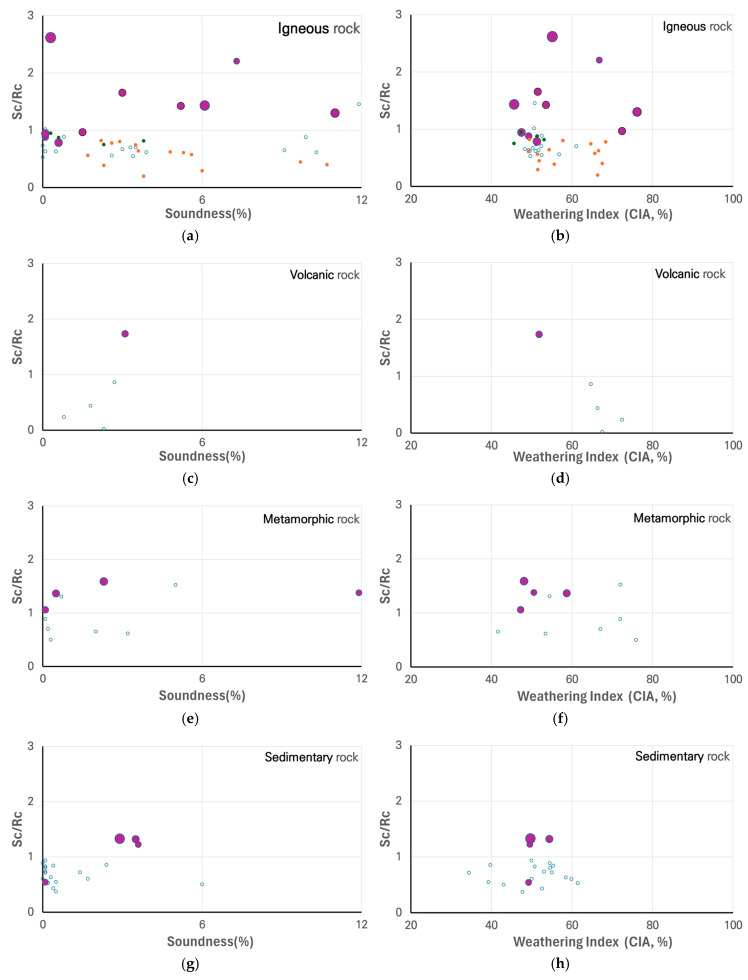
Comparison of soundness–ASR and CIA–ASR trends in rock samples. (Small blue circles (°) represent non-reactive samples in the ASTM C 1260 test, while large red circles indicate potentially reactive sample).

Figure 5 presents a comparison of the soundness–ASR and CIA–ASR trends. The CIA and CWI values for most ASR-reactive igneous rock samples were below 60 (e.g., CIA = 47.5, CWI = 51.2, 0.13% mortar bar expansion for 23YD-MG04), indicating moderate weathering. However, some igneous ASR samples exhibited significantly higher values, exceeding 60 (e.g., CIA = 76.2, CWI = 98.2, 0.14% mortar bar expansion for 21AS-MG04), suggesting that high weathering indices do not necessarily correlate with reduced ASR potential. These trends are summarized in Table 4. This discrepancy may stem from variations in mineralogical composition—particularly the presence of microcrystalline or strained quartz—which are not fully reflected by CIA values alone. Further investigation is recommended to clarify this relationship.

### 3.3. Summary of the Reactivity Trends and Anomalies

The results indicate that 20% of the 84 Korean aggregate samples show a propensity for ASR, in contrast to the historical classification of Korea as a region with minimal ASR risk. This result is supported by the comprehensive dataset for 17 of the 21 samples (Table 4), which are all classified as having potentially deleterious expansion in chemical and mortar bar expansion tests. These samples include a broad range of rock types, including granite (e.g., 0.17% mortar bar expansion and Sc = 106, Rc = 40.5 chemical reactivity for sample 22MJ-MG05), gneiss (e.g., 0.14% mortar bar expansion and Sc = 138.6, Rc = 106.5 chemical reactivity for 21AS-MG04), schist (e.g., 0.013% mortar bar expansion and 0 Sc= 128.3, Rc = 92.6 chemical reactivity for 21KJ-MG01), tuff (e.g., 0.16% mortar bar expansion and Sc = 168.9, Rc = 138.1 chemical reactivity for 21KJ-MG14), phyllite (e.g., 0.13% mortar bar expansion and Sc = 84, Rc = 52.9 chemical reactivity for 24JP-MG05), shale (e.g., 0.12% mortar bar expansion and Sc = 102.6, Rc = 77.8 chemical reactivity for 24ES-MG05). The verification of measurable expansion, well over the ASTM C 1260 threshold of 0.10% at 14 days, and detectable silica dissolution in the chemical tests suggest these aggregates are likely to undergo ASR-induced deterioration under standard conditions.

Furthermore, ~7% of the igneous rock samples show discrepancies between the ASR chemical test and mortar bar test, suggesting that the wrong choice of test might lead to significant errors. Mortar bar expansion data were reported for four samples, despite chemical reactivity results indicating no ASR potential reactivity or deleteriousness in the tested samples (23YD-MG01, granite; 23YD-MG04, granite; 22IC-MG04, granite; 24GJ-MG08, andesite). Among these samples, the granite exhibited relatively lower stability than the average, showing relatively low values of NAGpH, NAPP, and CIA. This indicates fewer cracks and pores that might form by natural weathering or artificial processing. Consequently, these rock characteristics may have influenced the results of the chemical ASR test. In contrast, sample 22PC-MG24 (marble) was identified as having deleterious expansion in the chemical test but non-deleterious expansion in the mortar bar test, likely due to the dissolution of calcium ions during the former. The absence of these data introduce uncertainty, particularly for andesite (sample 0.11% expansion and Sc = 4.6, Rc = 2.6 chemical reactivity for 24GJ-MG08), which is known to contain reactive amorphous silica. This omission precludes a definitive assessment of their ASR potential, because expansion tests are critical for confirming reactivity in the presence of alkali hydroxides, particularly given the limitations of ASTM C 289, which may underestimate the reactivity due to interference from minerals such as talc or zeolite, and therefore might not be indicative of ASR reactivity.

Beyond the direct ASR metrics, the supplementary tests also identified additional complexities. Soundness values (0.1–11.9% mass loss; Table 4) indicate the physical integrity is robust for most samples, with 16 of the 21 samples having values of <5% (e.g., 23BA-MG03 = 0.1%; 23KJ-MG14 = 3.6%). However, outliers such as samples 21ICN-MG08 (schist; 11.9%) and 21AS-MG04 (gneiss; 11%) approach or exceed the 12% threshold, signaling potential susceptibility to physical breakdown. Such degradation could increase the effects of ASR by increasing the surface area exposure of reactive silica, even if the initial reactivity appears low. The acid drainage parameters are also variable.

The NAGpH values ranged from 2.4 (sample 22PC-MG20) to 8.14 (sample 22PC-MG24), with most samples (16 of 21) exceeding the 4.5 threshold for acid generation risk, indicating chemical stability under acidic conditions. Conversely, NAPP values varied from –888.4 kg H_2_SO_4_/ton (sample 22PC-MG24) to 109.8 kg H_2_SO_4_/ton (sample 24GS-MG02), with the latter positive outlier indicating the potential for substantial acid production. This anomaly in sample 24GS-MG02 (gneiss) suggests a latent chemical reactivity that might be triggered under specific environmental conditions (e.g., prolonged exposure to ASR) and deviates from the predominantly acid-neutralizing behavior observed in the other samples (e.g., 21ICN-MG08: –32.75 H_2_SO_4_/ton; 24JP-MG05: –134.3 H_2_SO_4_/ton).

The CIA values ranged from 1.3 (sample 22PC-MG24) to 76.2 (sample 22AS-MG24), with some samples (6 of 21) exceeding the threshold value of 55 for weathering risk. The granite samples generally exhibit moderate alteration (e.g., CIA = 47.5 and CWI = 51.2 for 23YD-MG04; CIA = 55.1 and CWI = 74.1 for sample 22MJ-MG05), while the gneiss samples have a broader range of values, from slightly weathered (e.g., CIA = 59.1 and CWI = 66.4 for 22DDC-MG03) to highly altered (e.g., CIA = 76.2 and CWI = 98.2 for 21AS-MG04). These indices suggest that the weathering extent varies significantly, which potentially affects the silica availability for ASR, although no direct correlation with reactivity is evident from the limited dataset. Collectively, these findings confirm the low ASR risk of Korean aggregates, but highlight heterogeneity in the durability characteristics of the aggregates, as indicated by the supplementary tests, and some specific anomalies, including the test results for 24GS-MG02 (NAPP and CIA) and 21ICN-MG08 (soundness). These results indicate that expansion might occur due to factors other than ASR. These limitations suggest that interpretations should be made with care and that further investigations are needed.

## 4. Discussion

### 4.1. Mechanisms of ASR in Korean Aggregates

The significant ASR reactivity of some of the 84 Korean aggregate samples is consistent with the chemical and physical mechanisms of ASR, whereby reactive silica (Si[OH]_4_) interacts with alkali ions (Na^+^ or K^+^) to form an expanding gel, modulated by calcium hydroxide (Ca[OH]_2_). This process is controlled by the availability of reactive silica, alkalis, and moisture [16]. Olajide et al. [24] reported that moisture and temperature are critical drivers of ASR kinetics, suggesting that controlled test conditions (e.g., 80 °C for 14 days as per ASTM C 1260) may not fully replicate field conditions under which prolonged moisture exposure could activate latent reactivity.

The outlier sample 24GS-MG02, with a high NAPP value (109.8 kg H_2_SO_4_/ton), indicates potential chemical instability, which is possibly linked to accessory minerals or weathering (CIA = 72.4 and CWI = 89.2), which could enhance the silica solubility under alkaline conditions, although expansion was observed (Table 4). However, even after excluding external disturbances, the potential for ASR was observed in a significant number of samples. The samples are dominated by quartz-rich lithologies (e.g., granite and gneiss), as inferred from XRD data (Table 2). Some of the granite and gneiss samples are therefore likely to contain amorphous silica in addition to quartz, which exhibits high reactivity [17,21].

### 4.2. Comparison with Global Studies

The ASR potential of some Korean aggregates is not significantly different from other global cases, where reactive aggregates, particularly volcanic (e.g., andesite) or recycled types, pose significant challenges [2,5]. The granite and gneiss samples used in this study yielded results consistent with those of Yun et al. [9], who investigated other Korean rocks [15], but differ from international studies reporting an expansion of 0.3–0.5% for quartzites or cherts [25]. Santos et al. [5] noted that recycled concrete aggregates (RCAs) often exhibit increased ASR risk due to residual reactive silica. This concern does not apply to the Korean samples examined in this study, as they were sourced from natural rock materials. Nevertheless, if potentially reactive rocks are recycled as RCAs, the risk of ASR could be exacerbated.

Globally, ASTM C 1260 defines aggregate expansion greater than 0.20% as deleterious. This threshold was not exceeded by these samples, in contrast to reactive aggregates tested elsewhere (e.g., RILEM AAR-2 data and Bakera and Alexander [19]). However, expansion within the range of 0.1–0.2%, which is classified as potentially deleterious, was observed in various rock types. The use of ASTM C 1260, despite its limitations, is consistent with previous Korean studies, but may underestimate the reactivity as compared with modern modeling approaches [26]. Given the limitations of short-term tests, a two-stage mitigation strategy should be adopted, involving (1) screening for aggregate reactivity, and (2) validating the expansion behavior of concrete mixtures using long-term test methods.

The weathering indices (CIA = 1.3–76.2; CWI = 1.3–98.2) show no direct correlation with ASR, contrasting with the results of Šachlová et al. [27] who documented a link between alteration and reactivity. The high weathering index value for sample 24GJ-MG06 (andesite) limits our ability to explain the characteristics of foreign volcanic aggregates in terms of amorphous silica reactivity, given the small sample size [24].

### 4.3. Implications for Concrete Durability

The potentially deleterious outcomes of ASR suggest Korean concrete infrastructure is potentially vulnerable in terms of long-term durability performance, which is consistent with its historic resilience despite some late-1990s incidents [9]. However, Mohammadi et al. [3] cautioned that even minimal reactivity can degrade concrete tensile strength over time, which is a risk amplified by soundness outliers (e.g., samples 21ICN-MG08 and 21AS-MG04) that may expose reactive surfaces in moist environments such as highways.

Olajide et al. [24] highlighted the role of moisture in sustaining ASR, implying that humid coastal regions of Korea (e.g., Ansan) could exacerbate the latent risks of highly weathered samples (e.g., sample 21AS-MG04). The anomalous NAPP value of sample 24GS-MG02 suggests potential long-term chemical instability, which might form gels under alkali-rich conditions. Mohamed [28] noted that alkali-activated systems, while durable, may exhibit high shrinkage, which is an important consideration if Korean aggregates were paired with such binders. Recycled aggregates were not tested in this study, but have additional durability risks due to variable reactivity [29] and are relevant to the sustainability goals of Korea. These findings highlight the need for monitoring beyond ASR tests, including acid drainage tests, given the climatic diversity in Korea.

### 4.4. Mitigation Strategies

The low, but variable, ASR risk necessitates targeted mitigation to ensure concrete longevity. The use of the ASTM C 1260 test is consistent with Islam and Akhtar for its reliability [6]. Adopting ASTM C 1260, as endorsed by them for its reliability, can help address the data gaps for samples for which mortar bar expansion data are currently unavailable (e.g., 23YD-MG01 and 24GJ-MG06). Although ASTM C 1260 is a fast and useful screening tool, it is not sufficient for definitive acceptance or rejection. Aggregates identified as potentially reactive should undergo long-term confirmation testing, such as ASTM C 1293, and in the U.S., a transition is underway from ASTM C289 to more reliable methods such as T-FAST [30].

Shi and Lothenbach [17] advocated for the use of alumina-rich SCMs (e.g., metakaolin) and lithium salts to suppress gel formation, and Bakera and Alexander [19] confirmed the efficacy of metakaolin in reducing expansion by up to 89% in reactive mixes, which would be applicable to outliers such as sample 24GS-MG02. Gillott and Wang [20] suggested limiting the alkali content, which is a practical measure for soundness-compromised samples (e.g., 21ICN-MG08), while moisture control using sealants is critical in humid regions [2,24]. For recycled aggregates, which are increasingly used in Korea, Santos et al. [5] and Nikmehr and Al-Ameri [31] and Wongpaun et al. [32] highlighted the ASR risks and proposed the use of geopolymer concrete as a sustainable option, although Mohamed [28] noted challenges such as shrinkage and carbonation in alkali-activated slag systems. Piccinali et al. [29] emphasized the need for RCA quality control, suggesting pre-treatment (e.g., thermal processing) could reduce the reactivity, which is a strategy adaptable to Korea. Demolition and replacement are often the only remediation option for structures affected by ASR. In Korea, significant costs are also being incurred for the demolition and reconstruction of ASR-affected highways [15]. These measures that integrate global innovations with local findings could enhance concrete durability while supporting sustainable construction.

## 5. Conclusions

The ASR potential of 84 aggregate samples sourced from various geological regions across the Republic of Korea was assessed. Some granite, gneiss, schist, and tuff samples exhibit a risk of deleterious expansion as a result of ASR.

Based on the soundness, acid drainage, and weathering index test results, factors unlikely to be caused by ASR were ruled out; however, reactivity was still confirmed in granite, gneiss, schist, and tuff samples. No samples exhibited reactivity at a severely deleterious level (>0.2% expansion). This observed stability is attributed to the prevalence of crystalline quartz within the aggregates, coupled with the geological characteristics of Korea, such as minimal tectonic stress and scarce volcanic activity, which limits the formation of reactive silica polymorphs [17,18]. Supplementary tests, including soundness and acid drainage analyses, largely confirmed the physical and chemical stability of the aggregates. However, high weathering index values or acid-sensitive samples indicate the need to interpret ASR reactivity results with caution. These findings confirm the suitability of Korean aggregates for concrete production, providing a robust basis for their continued use in national infrastructure projects.

To ensure long-term concrete durability, several practical measures are recommended. Currently, Korea does not have an accelerated ASR testing standard such as ASTM C 1260. A comparable Korean testing standard involves measuring length changes over a 6-month period under natural exposure conditions. To ensure the timeliness required in the construction industry, it is recommended that ASTM C 1260 be adopted as a standard test method. For aggregates displaying borderline reactivity or elevated weathering index values, The incorporation of SCMs, such as metakaolin, is proposed to suppress ASR gel formation and mitigate expansion [19]. However, the high cost of metakaolin and other SCMs limits their widespread use in Korea, particularly in a context marked by low awareness of ASR risks, a stagnant cement industry, and the lack of supportive policy frameworks. In addition, controlling the cement alkali content and implementing moisture management strategies, particularly in humid coastal regions, are critical to minimizing the ASR risks [7]. Policymakers are urged to revise the national standards to be consistent with global concrete durability benchmarks, thereby facilitating the integration of these practices.

While this study provides a basis for understanding the ASR potential of Korean aggregates, further research is warranted. Long-term field monitoring of concrete structures constructed with these aggregates, especially in high-humidity environments, is essential to validate the laboratory findings. Given the increasing emphasis on sustainable construction practices, investigating the ASR behavior of RCA in Korean conditions is required [5]. Investigating the efficacy of advanced mitigation strategies in Korea, such as lithium-based admixtures or geopolymer concrete, could yield innovative solutions applicable to both virgin and recycled aggregates [29,31]. Finally, analysis of the correlation between weathering indices and ASR reactivity could enhance predictive models of aggregate stability and improve the precision of durability assessments. In summary, this study confirmed that the ASR reactivity of Korean aggregates cannot be considered completely safe. Implementing the recommended testing and mitigation strategies, along with the proposed future research directions, will enable Korea to maximize the durability and sustainability of its concrete infrastructure.

## Figures and Tables

**Table 1:** Rock types of the 84 aggregate samples tested for ASR potential.

Rock Classification			Samples
Igneous	Granite	Granite	17
		Biotite granite	2
		Porphyritic granite	1
		Gneissic granite	2
	Gneiss	Gneiss	4
		Banded gneiss	5
		Porphyroblastic gneiss	2
		Granite gneiss	2
		Granitic gneiss	4
		Migmatitic gneiss	2
	Diorite	Diorite	2
		Granodiorite	1
	Porphyry	Granite porphyry	1
		Quartz porphyry	1
Volcanic	Andesite	Andesite	1
		Microcrystalline andesite	1
		Tuffaceous andesite	1
		Andesite rocks	2
Metamorphic	Schist	Schist	5
		Siliceous schist	1
	Phyllite	Phyllite	1
		Sand-rich phyllite	1
		Calcite-rich phyllite	1
	Quartzite	Quartzite	1
	Slate	Slate	1
Sedimentary	Sandstone	Sandstone	5
		Sandstone rocks	1
	Tuff	Tuff	6
	Shale	Shale	4
		Shale rocks	1
	Sedimentary Rock	Granitic sedimentary rocks	1
		Sand-rich sedimentary rocks	1
		Metamorphic sedimentary rocks	1
	Siltstone	Siltstone	1
	Marble	Marble	1
Total			84

**Table 2:** Mineralogical compositions of the aggregate samples determined by XRD analysis.

Sample Name	Rock Type	Composition Minerals
24JP-MG05	Phyllite	Biotite, Ca-plagioclase, Quartz
24GS-MG02	Gneiss	Muscovite, Quartz, Pennantite, Albite, Biotite
24ES-MG05	Shale	Muscovite, Quartz, Albite, Calcite, Pennantite, Hematite
23NS-MG06	Schist	Quartz, Muscovite, Biotite, Pennantite, Calcite
23BA-MG03	Granite	Albite, Quartz, Microcline, Muscovite, Pennantite, Biotite, Magnetite, Calcite
22PC-MG24	Marble	Calcite, Dolomite, Serpentine
22PC-MG20	Granite	Plagioclase, Quartz, K-feldspar, Muscovite, Biotite
22PC-MG19	Diorite	Plagioclase, Quartz, Chlorite, Biotite, Hornblende
22MJ-MG05	Granite	Plagioclase, Quartz, K-feldspar, Muscovite, Chlorite
22DDC-MG03	Gneiss	Plagioclase, Quartz, Muscovite, Biotite
21YP-MG06	Gneiss	Plagioclase, Quartz, Pennantite, Biotite, Garnet
21KJ-MG20	Tuff	Plagioclase, Quartz, K-feldspar, Chlorite, Epidote
21KJ-MG14	Tuff	Plagioclase, Quartz, K-feldspar, Muscovite, Hematite, Chlorite
21KJ-MG01	Schist	Quartz, Plagioclase, Muscovite, K-feldspar
21ICN-MG08	Schist	Biotite, Plagioclase, K-feldspar, Quartz
21CJ-MG06	Gneiss	K-feldspar, Pennantite, Magnetite, Hematite, Biotite
21AS-MG04	Gneiss	Quartz, Muscovite
24GJ-MG08	Andesite	Quartz, Albite, Pennantite, Muscovite, Orthoclase, Calcite
23YD-MG04	Granite	Quartz, Orthoclase, Hornblende, Biotite, Pennantite, Magnetite
23YD-MG01	Granite	Albite, Quartz, Hornblende, Microcline, Pennantite
22IC-MG04	Granite	Plagioclase, Quartz, K-feldspar, Muscovite, Pennantite, Biotite

Sample names are composed of the sampling year, location, type (MG = mountain gravel), and a unique identifier.

**Table 3:** Chemical compositions of the aggregate samples determined by XRF analysis.

Sample Name	SiO_2_	Al_2_O_3_	Fe_2_O_3_	CaO	MgO	K_2_O	Na_2_O	TiO_2_	MnO	P_2_O_5_	Lg. Loss
24JP-MG05	59.9	15.35	6.38	6.58	3.95	3.27	0.86	0.73	0.09	0.15	1.74
24GS-MG02	59.03	18.82	7.35	0.3	2.91	4.61	1.06	0.81	0.07	0.05	3.84
24ES-MG05	61.82	14.21	5.56	3.34	2.88	3.44	1.52	0.8	0.06	0.16	5.91
23NS-MG06	69.41	11.95	3.46	5.23	3.36	3.12	0.46	0.65	0.07	0.13	1.61
23BA-MG03	72.04	13.85	1.76	1.64	0.36	3.95	3.92	0.29	0.02	0.08	1.43
22PC-MG24	5.49	1.24	0.5	45.88	7.07	0.29	0.02	0.07	0.02	0.01	38.53
22PC-MG20	76.18	13.08	0.91	0.71	0.05	4.78	3.65	0.06	0.04	0.02	0.32
22PC-MG19	50.09	17.98	10.36	8.07	4.45	2.14	2.83	1.64	0.15	0.32	1.06
22MJ-MG05	78.55	11.45	1.49	0.32	0.27	4.91	2.14	0.05	0.03	0.04	0.65
22DDC-MG03	64.49	16.79	5.54	3.0	1.96	2.90	2.92	0.49	0.13	0.08	0.77
21YP-MG06	68.98	13.97	5.46	3.07	1.87	1.26	3.29	0.53	0.08	0.1	0.96
21KJ-MG20	71.81	13.45	2.86	1.66	0.61	3.85	4.04	0.29	0.1	0.09	0.86
21KJ-MG14	72.77	13.04	2.77	0.79	0.28	4.81	4.09	0.2	0.09	0.04	0.64
21KJ-MG01	78.31	10.53	2.47	1.3	1.13	3.76	0.68	0.34	0.04	0.06	1.64
21ICN-MG08	59.81	15.4	7.97	3.86	2.23	3.71	3.41	1.53	0.11	0.66	0.89
21CJ-MG06	64.97	15.15	6.17	1.9	1.01	5.41	3.3	0.78	0.1	0.2	0.57
21AS-MG04	95.5	2.64	0.33	0.03	0.06	0.72	0.01	0.05	0.01	0.01	0.61
24GJ-MG08	68.84	13.56	5.1	2.02	2.14	2.44	2.82	0.91	0.03	0.05	1.37
23YD-MG04	65.52	14.99	4.38	4.81	2.04	2.11	3.55	0.45	0.09	0.12	1.21
23YD-MG01	60.83	15.91	6.65	4.32	2.59	1.86	4.24	0.71	0.13	0.19	1.78
22IC-MG04	65.52	14.99	4.38	4.81	2.04	2.11	3.55	0.45	0.09	0.12	1.21

**Table 4:** Aggregate reactivity assessment results based on ASR and related properties.

Sample Name	Alkali Aggregate Reaction		Soundness	Acid Rock Drainage		Weathering Index	
	Chemical	Mortar Bar Expansion (%)	(%)	NAGpH (pH)	NAPP (kg H_2_SO_4_/t)	CIA	CWI
24JP-MG05	potentially deleterious	0.13	2.3	7.52	−134.3	48.1	54.1
24GS-MG02		0.12	1.6	4.3	109.8	72.4	89.7
24ES-MG05		0.12	3.5	7	−8.7	54.4	63.4
23NS-MG06		0.11	0.1	5.75	−1.75	47.3	54.6
23BA-MG03		0.1	0.1	6.78	−2.48	50.6	60
22PC-MG24		0.06	0.5	8.14	−888.4	1.3	1.3
22PC-MG20		0.1	7.3	2.4	−0.04	66.8	90.8
22PC-MG19		0.07	2.3	2.83	−26.78	43.3	45.9
22MJ-MG05		0.17	0.3	3	−4.36	55.1	74.1
22DDC-MG03		0.1	0.8	3.02	−12.48	59.1	66.4
21YP-MG06		0.08	5.2	6.12	−17.69	53.5	56.5
21KJ-MG20		0.1	2.9	5.8	−10.24	49.7	58.7
21KJ-MG14		0.16	3.6	6.49	−7.79	49.6	61.8
21KJ-MG01		0.12	0.5	6.8	−17.75	58.7	75.9
21ICN-MG08		0.1	11.9	6.2	−32.75	50.6	58.2
21CJ-MG06		0.12	3	5.12	−11.01	51.5	64.3
21AS-MG04		0.14	11	4.93	0.18	76.2	98.2
24GJ-MG08	considered potentially deleterious	0.11	3.1	4.56	2.6	54.6	58.1
23YD-MG04		0.13	0.1	5.86	−6.43	47.5	51.2
23YD-MG01		0.11	0.1	6.11	−5.99	49.3	52.5
22IC-MG04		0.13	0.6	6.06	−12.43	51.3	61.3

## Data Availability

The original contributions presented in this study are included in the article. Further inquiries can be directed to the corresponding author.
